# Longitudinal Analysis of P100 Wave Amplitude and Latency in Multiple Sclerosis: A 19-Year Retrospective VEP Study

**DOI:** 10.3390/diagnostics15101189

**Published:** 2025-05-08

**Authors:** Manuela Andreea Ciapă, Vlad Constantin Donica, Claudia Florida Costea, Camelia Margareta Bogdănici

**Affiliations:** Department of Ophthalmology, Faculty of Medicine, University of Medicine and Pharmacy “Grigore T. Popa”, University Street, No. 16, 700115 Iasi, Romania; manuela-andreea_v_tanasachi@d.umfiasi.ro (M.A.C.); claudia.costea@umfiasi.ro (C.F.C.); camelia.bogdanici@umfiasi.ro (C.M.B.)

**Keywords:** multiple sclerosis, visual evoked potentials, latency, amplitude, p100 wave complex

## Abstract

**Background:** The diagnosis of multiple sclerosis (MS) relies on identifying neurological signs and symptoms, supported by evidence of central nervous system (CNS) dissemination of lesions across time and space. The visual pathway is commonly involved in MS, with a frequent involvement of optic neuritis (ON) episodes. Our study aims to assess the relationship between neuronal damage and optic nerve demyelination by analyzing the latency and amplitude of the p100 wave complex using visual evoked potentials (VEPs). **Methods:** We conducted a retrospective longitudinal study, analyzing VEP records of 15 patients with recurrent remissive MS at baseline, 5, 10, 15, and 19 years. **Results:** In 30 eyes we observed an increase in p100 wave latency at 5-years by 14.35 ± 4.47 ms (*p* = 0.003), at 10-years by 19.26 ± 4.87 ms (*p* < 0.0005) and a decrease in amplitude by 2.29 ± 0.52 mV (*p* < 0.0005) when comparing to baseline values. At 15-years, 24 eyes presented an increase in latency of 31.39 ± 7.8 ms (*p* = 0.001) and a decrease in amplitude of 2.51 ± 0.6 mV (*p* < 0.0005) compared to baseline, while at 19-years, 10 eyes presented an increase in p100 wave latency of 53.45 ± 18.42 ms (*p* = 0.018) and a further decrease in amplitude of 4.06 ± 1.32 mV (*p* = 0.014). We found correlations between the p100 wave latency and amplitude at baseline, 15-year, and 19-year follow-ups, increasing from a low negative (r = −0.43) to medium negative (r = −0.502) and finally high negative (r = −0.906) correlation. **Conclusions:** VEPs have long been acknowledged for their ability to detect both clinical and subclinical lesions in MS cases. Our study offers new insight into the relationship between demyelination and axonal degeneration observed when analyzing the latency and amplitude of the p100 wave complex during VEP in a longitudinal analysis.

## 1. Introduction

Multiple sclerosis (MS) is a chronic inflammatory disease of the central nervous system (CNS), characterized by demyelination and neurodegeneration [1]. There is an increasing rate of the incidence and prevalence of the disease in both developed and developing countries [2]. MS is the most common cause of non-traumatic disability among young adults [3]. The diagnosis of multiple sclerosis is based on neurological signs and symptoms, along with evidence of the dissemination of CNS lesions in both time and space. Symptoms during a relapse may resolve after days or weeks or lead to residual deficits. The visual pathway is frequently affected in MS, with episodes of optic neuritis (ON) often present even in the early stages of the disease [4]. It is estimated that approximately 25% of MS patients experience an episode of ON as an initial manifestation of the disease, while up to 70% experience such an episode during the course of the illness [5]. The typical demyelinating form of ON is characterized by unilateral vision loss developing over hours to up to two weeks, periorbital pain, particularly during eye movement, and dyschromatopsia [6].

Depending on the location of the inflammatory lesion, ON can be classified as retrobulbar, in which the optic disc appears normal during fundoscopy or as papillitis, when there are visible signs of optic disc edema. Optical coherence tomography (OCT) has proven useful in providing structural biomarkers for monitoring MS activity and severity by measuring the thickness of the macular ganglion cell layer thickness and of the peripapilar retinal nerve fiber layer. However, during ON, OCT can mostly identify inflammatory lesions near the optic disc, while MRI can be used to assess cases with retrobulbar inflammation [7].

In order to assess functional impairment during ON, visual evoked potentials (VEPs) have long been recognized as capable of detecting clinical and subclinical lesions in MS cases, with their results being included as diagnostic criteria in MS [8]. VEPs provide information regarding lesions along the optic path from the eyeball to the occipital cortex [9].

Halliday et al. described a reduction in visual evoked potentials (VEPs) during the acute phase of neuritis. Subsequently, as vision recovers, a delayed VEP with reduced amplitude can be recorded. The amplitude of the VEP progressively increases in parallel with the improvement of visual acuity. According to Halliday et al., the prolonged VEP latency does not return to normal. They concluded that once stabilized, the latency recorded in a given patient appears to remain unchanged unless a new episode of optic neuritis occurs, which will further increase the delay [10].

Demyelination, the hallmark of multiple sclerosis, can be observed in the visual system, where lesions are common and manifest as delayed nerve conduction, highlighted by visual evoked potentials (VEPs) [11]. Most frequently, the stimulus used for this test is a checkerboard pattern with alternating squares (pattern VEP) [12]. In standard VEP evaluations, three waves can be identified: N75, P100, and N145. The P100 wave, a positive potential occurring at approximately 100 ms, is used clinically for interpretation of the results [13]. Alterations in VEP latencies with patterned stimulation are considered one of the most significant electrophysiological features observed in MS patients. The electrophysiological effects of demyelination may improve, worsen, or remain stable [14].

Modifications of the P100 wave complex in eyes without a history of optic neuritis in MS patients represent a marker of systemic neurodegeneration, highlighting the importance of VEP in monitoring the activity of the neurodegenerative condition [15].

Our study aims to evaluate the relationship between neuronal damage and demyelination of the optic nerve, hallmark lesions in MS patients with positive ON history, by analyzing the p100 wave latency and amplitude over the course of 19 years using VEPs.

## 2. Materials and Methods

We conducted a retrospective study, analyzing the medical records of MS patients under the care of the Neurology Clinic at the Rehabilitation Hospital in Iași, Romania, from 2004 to 2024. The study design and protocol were performed according to the tenets of the Declaration of Helsinki for research involving human subjects and approved by the Ethics Committee of the Rehabilitation Hospital Iasi, Romania (No. 13/6 October 2020). Subjects have signed an informed consent prior to examination.

Our analysis identified 15 patients, with a definitive recurrent remissive MS diagnosis established based on clinical, imaging, and biological evaluations. The initial assessment included an ophthalmological and neurological consultation, the recording of visual and auditory evoked potentials, and psychological testing. The subjects were under immunomodulatory therapy, and the monitorization of VEP was required as part of the National MS treatment program.

The VEP recordings were performed using a NIHON KOHDEN device, with a checkerboard-patterned stimulation, adhering to international recommendations: monocular stimulation, a stimulus rate of 1 cycle/second, a contrast of 0.5 (corresponding to a minimum 3:1 ratio between maximum and minimum illumination), and a visual angle (defined as the size of a single bright or dark element) of 10 min. The amplifier filters were set to 1–30 Hz. The recorded parameters included the P100 wave latency (measured in milliseconds) and the P100 wave amplitude (measured as the peak-to-peak value between the N75 and P100 waves, in microvolts).

Our analysis identified VEP reports that have been gathered in the following manner, 30 eyes at baseline, at the 5-year follow-up (FU), and at the 10-year FU, while 24 eyes had 15 years of FU, and 10 eyes reached 19 years of FU.

### Statistical Analysis

Statistical analysis was performed using SPSS statistical package, version 26.0. A paired-samples *t*-test was used to determine whether there was a statistically significant mean difference in the p100 wave latency and amplitude between baseline and at the 5-year, 10-year, 15-year, and 19-year landmarks. Data are mean ± standard deviation, unless otherwise stated. Outliers were detected that were more than 1.5 box-lengths from the edge of the box in a boxplot. Inspection of their values did not reveal them to be extreme, and they were kept in the analysis. The assumption of normality was violated in the 15-year latency and 19-year amplitude datasets.

A Pearson’s correlation was run to assess the relationship between latency and amplitude of the p100 wavelength at baseline and at the 5-year, 10-year, 15-year-, and 19-year landmarks. A level of *p*  <  0.05 was accepted as statistically significant.

## 3. Results

We analyzed 30 eyes at baseline and compared them with their FU at 5 and 10 years, 24 eyes reached 15 years of FU, while 10 were evaluated for 19 years. The statistical analysis results can be seen in Table 1. Patient age had an average of 34.6 years with a range between 22 and 47 years at baseline.

At the 5-year FU, the p100 wave latency had a statistically significant increase by 14.35 ± 4.47 ms (*p* = 0.003), while the amplitude did not present statistically significant modifications (*p* = 0.123).

At the 10-year FU, we observed a statistically significant increase in latency by 19.26 ± 4.87 ms (*p* < 0.0005) and a decrease in amplitude by 2.29 ± 0.52 mV (*p* < 0.0005).

24 eyes reached 15 years of FU, having a statistically significant increase in the p100 wave latency of 31.39 ± 7.8 ms (*p* = 0.001) and a decrease in amplitude of 2.51 ± 0.6 mV (*p* < 0.0005) compared to baseline.

At 19-years of FU, 10 eyes presented a statistically significant increase in the p100 wave latency of 53.45 ± 18.42 ms (*p* = 0.018) and a further decrease in amplitude of 4.06 ± 1.32 mV (*p* = 0.014).

**Figure 1:**
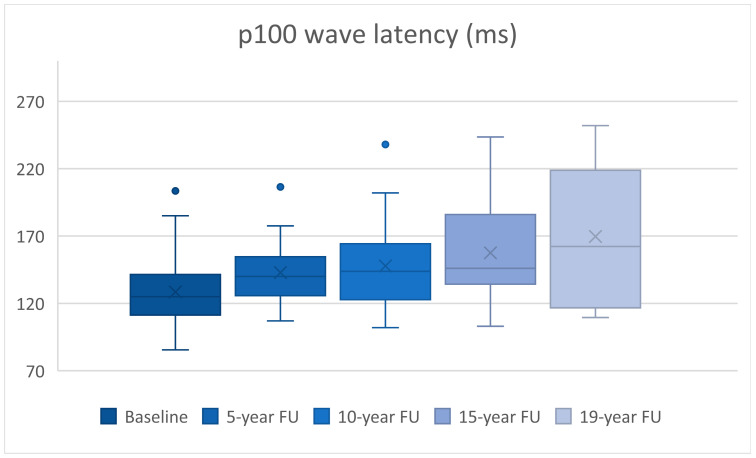
p100 wave latency (ms) increase over time.

**Figure 2:**
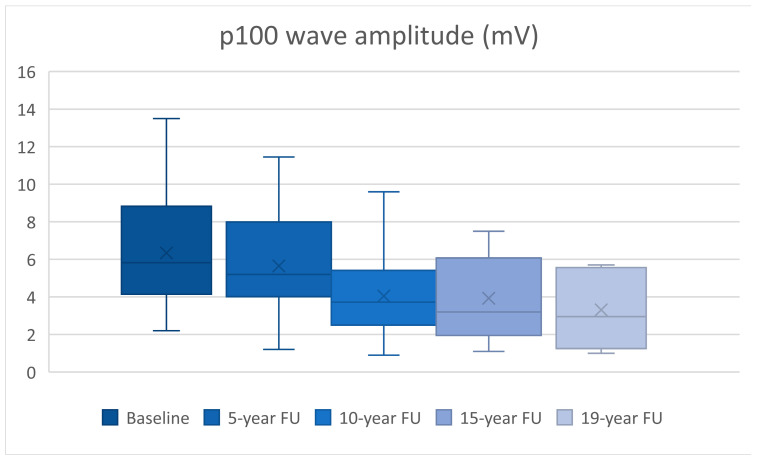
p100 wave amplitude (mV) decrease over time.

A steady increase in latency can be observed during every follow-up (Figure 1), whilst a decrease in amplitude occurs at the same time (Figure 2).

We found statistically significant correlations between the p100 wave latency and amplitude at baseline and the 15-year and 19-year follow-ups. The 5- and 10-year FUs presented an increasing negative correlation but did not reach statistical significance. Values and statistical significance can be seen in Table 2.

**Figure 3:**
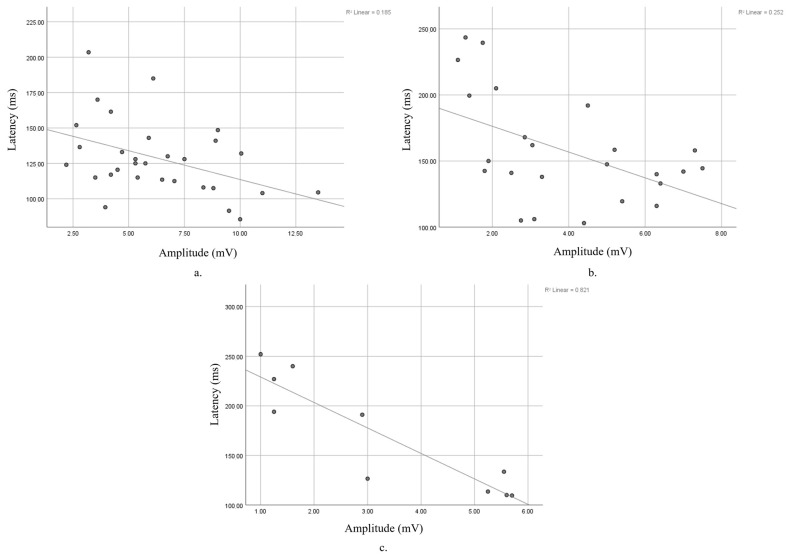
Correlation between amplitude and latency (**a**) at baseline; (**b**) at 15-year FU; (**c**) at 19-year FU.

The relationship between latency and amplitude rose from a low negative (r = −0.43) at baseline (Figure 3a) to medium negative at the 15-year FU (r = −0.502) (Figure 3b), and finally reaching a high negative (r = −0.906) (Figure 3c) correlation.

## 4. Discussion

The earliest studies attempting to correlate evoked potential changes with pathological findings in MS were based on VEPs, as they were the first to provide a reliable diagnostic marker for MS. This was achieved through the recognition and identification of one of the initial manifestations of the demyelinating disease, ON, characterized by the distinctive VEP alteration in MS, an increased latency of the major positive component.

Multiple studies have reported an increased detection in optic nerve lesions using MRI, OCT, and VEPs after focusing their attention on the optic nerve based on the recommendations from the 2017 revised McDonald criteria [16]. Based on these aspects, the addition of optic nerve involvement increased the rate of MS diagnosis and, therefore, led to the recommended inclusion of the optic nerve as the fifth area of the central nervous system in the 2024 revised criteria [17].

Roldán et al. evaluated the risk of conversion from radiologically isolated syndrome to MS over the course of 2 years using MRI and multifocal VEP (mfVEP). They concluded that a reduction in interocular and monocular amplitude and an increased interocular latency presented a higher risk of clinical conversion, signaling the role of mfVEP in predicting potential MS onset [18]. Our results provide insights regarding alterations of visual function in MS patients over the course of 19 years.

Vecchio et al. identified an increase in latency of the p100 wave in MS eyes with no history of ON, demonstrating the capacity of VEP in analyzing neurodegeneration [19]. In pediatric MS patients, VEP has also been able to establish a prolonged p100 latency, detecting clinical and subclinical optic path damage [20].

Filgueiras et al. compared p100 wave latency and amplitude between MS and neuromylitis optica spectrum disorder (NMOSD) patients with a history of ON. They found an increased latency and normal amplitude of the p100 wave in MS patients, with a lower amplitude and preserved latency in NMOSD [21]. Our results in MS patients were similar at the 5-year FU, having an increase in latency with no statistically significant modification regarding amplitude, but longer follow-ups revealed a statistically significant change in amplitude as well an increase in latency that were aggravated over time.

In a multicenter analysis, Oertel et al. analyzed the p100 latency and OCT structural parameters in 293 eyes from 146 patients with a minimum of two follow ups at more than one year intervals. Their analysis found a correlation between an increase in p100 latency and GCIPL loss for the next 3 years, with a correlation between the decrease in pRNFL thickness and the increase in p100 latency at baseline, but not during follow-ups [15].

Ekayanti et al. investigated p100 wave modifications using VEPs in healthy patients, analyzing age, gender, weight, BMI, and visual acuity. They concluded that latency is unaffected by these parameters, while the amplitude presented a statistically significant decrease with age, with female patients having higher amplitude values compared to males. While a decrease in amplitude is a sign of age-related neurodegeneration, significant differences appeared after 61 years of age. The higher values in women could be linked with a smaller head circumference and hormone protection [22]. Research into variations of retinal hemodynamics provided insight into the protective role of sex hormones in maintaining retinal structural and circulatory homeostasis [23,24]. Due to the retrospective nature of our research, we lack disability score data.

While VEPs have long been used in evaluating the visual path, recent data have highlighted the need for posterior visual path analysis when interpretating VEP latency in MS patients [25]. Papadopoulou et al. confirmed these aspects, identifying a strong correlation between p100 latency and lateral geniculate nuclei volume in MS patients with ON history, suggesting that synaptic damage at this level together with axonal degradation could be responsible for the increase in latency [26].

Following an acute ON episode, numerous immunomodulatory molecules are activated in order to limit inflammation, begin repair, and trigger oligodendrocyte-mediated remyelination. Understanding these molecular mechanisms could help with patient treatment by offering new agents that can improve patient prognosis [27]. Axonal function is disrupted due to myelin loss, and depending on the severity and duration of inflammation, axons may eventually degenerate. Inflammatory cytokines, enzymes, and nitric oxide, produced by activated immune cells, have direct cytotoxic effects and can damage axons. After inflammation subsides, surviving axons may undergo remyelination, but inflammatory lesions result in neuronal cell death, axonal loss, and gliosis [28].

Reactive gliosis, demyelination, and axonal death are all symptoms of inflammation mediated by activated microglia, monocyte-derived macrophages, and CD4+ and CD8+ T lymphocytes. Myelin-producing oligodendrocytes (MPOs) and oligodendrocyte precursor cells (OPCs) are targeted by pro-inflammatory cytokines and cytotoxic substances, leading to apoptosis and exacerbating axonal demyelination. Mature OPCs that have not undergone demyelination are unable to generate new myelin sheaths. Consequently, the migration and regeneration of oligodendrocytes from precursor cells are essential for remyelination. Retinal ganglion cells undergo retrograde degeneration as a result of these acute inflammatory lesions affecting the afferent visual pathway [29].

In MS, remyelination by oligodendrocytes has also been described, occurring primarily at the edges of newly formed plaques. In a later phase, a large number of microglial phagocytes infiltrate the lesion, while astrocytes within and around the lesion increase in both number and size. Older lesions contain dense fibro-glial tissue, which is relatively acellular, with sparse perivascular lymphocytes and macrophages, and are described as demyelinated sclerotic plaques [30].

The proliferative oligodendrocyte progenitor cell has been reported as the most efficient remyelinating cell in animal experiments for repairing demyelinating lesions, particularly those affecting the optic nerve [31]. The presence of a comparable population of OPC in the normal adult human white matter, as well as in acute and chronic MS lesions, may serve as a source for oligodendrocyte proliferation following demyelinating injuries in humans. Although the presence of OPC influences the eventual number of oligodendrocytes within a demyelinated lesion, the quantity of oligodendrocytes alone is not the sole factor required for effective remyelination.

Recurrent lesions of the demyelinated optic nerve can impair repair processes, leading to permanently demyelinated axons and remyelination failure [32]. This finding explains why remyelination is observed early in the course of MS but not in typical chronic MS lesions, which are more likely to have undergone multiple temporally distinct demyelination events [33]. Although beneficial, endogenous remyelination in ON and MS, in general, has inherent limitations.

Compared to normal axons, remyelinated axons exhibit thinner myelin sheaths and shorter internodal lengths [34]. Moreover, remyelinated axons demonstrate impaired axonal conduction velocities [35]. Ultimately, incomplete remyelination is a major factor contributing to the persistence of visual impairment in optic neuritis. The mechanism underlying conduction delays is likely more complex than a mere alteration in conduction along the plaque and is probably linked to a pathological modification of the myelin layer and early remodeling of axonal membrane properties. Additionally, the early increases in amplitude could be explained by the same progressive reorganization of conductive properties in previously blocked axons within extensively demyelinated regions [30]. The return of VEP latencies to values closer to normal may depend on the progressive modification of axonal properties or the gradual emergence of short remyelination foci. By forming short internodal regions, these foci may restore near-normal conduction in previously demyelinated areas.

While disease modifying therapies are expected to improve remyelination and hasten recovery after an inflammatory episode, and there have been promising results on animal models [36], current studies have not been able to provide evidence for this aspect, with VEPs showing no improvement in patients with DMT regarding remyelination or neurodegeneration [37].

The onset of neurodegeneration is variable. The observation that, following an episode of optic neuritis in which the amplitude of the P100 wave was reduced, subsequent improvements in amplitude can be recorded makes it challenging to pinpoint the exact start of neurodegeneration. OCT examination can be highly useful for this purpose. The amplitude of the P100 wave remains a valuable tool for assessing neurodegeneration; however, inter-individual variability in VEPs must be considered. Additionally, it is important to note that the NIHON KOHDEN device amplifies the initial signal by approximately 500,000 times.

Our patients presented ON history; therefore, we encountered difficulties in establishing baseline parameters in order to evaluate neurodegeneration. After establishing MS diagnosis and performing regular monitoring of disease activity and continuous immunomodulatory therapy, the increase in latency and decrease in amplitude of the p100 wave over 19 years suggest that chronic degeneration of the central nervous system occurs in a progressive manner.

The emergence of increasingly effective therapies for the treatment of MS has proven promising for a life without disability. For patients with RRMS, the average time to developing the secondary progressive form has been substantially extended. In the presence of effective treatment, relapses have been reduced both in intensity and in number. Despite these advancements, a silent progression independent of relapse was revealed, which had previously been masked by acute episodes and remissions [38]. This progression is in accordance with our findings regarding chronic degeneration in the absence of inflammatory episodes.

It is also important to consider that VEP results can be influenced by multiple factors related to the recording protocol, including patient cooperation, light intensity, pattern contrast, and stimulation rate [39]. To minimize these variables, visual evoked potentials should be recorded following international guidelines [40]. Additionally, establishing a normal variability range through the evaluation of a control group is necessary [41].

The limits of our study consist in the retrospective nature of our research, leading to a lack of data regarding clinical symptoms, disease severity score, number of inflammatory episodes, or radiological activity during the follow-up period and making correlations with other imaging modalities such as OCT unavailable. In addition, the low patient number included in this study could lead to errors during data analysis, with larger cohorts being required in order to confirm these changes in the general MS population.

Future directions should focus on longitudinal studies that correlate structural OCT data and VEP p100 wave amplitude and latency changes, while also evaluating the involvement of the posterior visual pathway degeneration mechanisms. In addition, machine learning and artificial intelligence algorithms could be used for better data analysis and to identify subclinical lesions.

## 5. Conclusions

The natural history of ON in MS unfolds all the more spectacularly the longer a patient is monitored using VEP recordings annually. The latter have long been acknowledged for their ability to detect both clinical and subclinical lesions in MS cases.

Our study offers new insight into the relationship between demyelination and axonal degeneration. While the latency of the p100 wave complex is increasing steadily over time with a statistical significance suggesting demyelination, the amplitude starts to have significantly lower values after 10 years of disease activity marking an important step in understanding the mechanism and timing of neurodegeneration in MS patients. Furthermore, the presence of a negative correlation between latency and amplitude of the p100 wave complex only at baseline and at distant follow-ups suggests that while neurodegeneration and demyelination may begin at the same time in the initial stages of MS, they follow separate courses during the disease, being affected by the neurodegenerative process in a similar pattern in advanced disease stages.

## Figures and Tables

**Table 1:** Paired sample *t*-test of p100 wave latency and amplitude.

	n	Latency (ms)	*p*	Amplitude (mV)	*p*
Baseline vs. 5-year FU	30	14.35 ± 4.47	*p* = 0.003	0.68 ± 0.43	*p* = 0.123
Baseline vs. 10-year FU	30	19.26 ± 4.87	*p* < 0.0005	2.29 ± 0.52	*p* < 0.0005
Baseline vs. 15-year FU	24	31.39 ± 7.8	*p* = 0.001	2.51 ± 0.6	*p* < 0.0005
Baseline vs. 19-year FU	10	53.45 ± 18.42	*p* = 0.018	4.06 ± 1.32	*p* = 0.014

**Table 2:** Pearson correlation between p 100 wave latency and amplitude.

	n	r	*p*
Baseline	30	−0.43	*p* = 0.018
5-year FU	30	−0.225	*p* = 0.232
10-year FU	30	−0.251	*p* = 0.181
15-year FU	24	−0.502	*p* = 0.012
19-year FU	10	−0.906	*p* < 0.0005

## Data Availability

The original contributions presented in this study are included in the article. Further inquiries can be directed to the corresponding author.

## References

[1] Kamm C.P., Uitdehaag B.M., Polman C.H. (2014). Multiple Sclerosis: Current Knowledge and Future Outlook. Eur. Neurol..

[2] Browne P., Chandraratna D., Angood C., Tremlett H., Baker C., Taylor B.V., Thompson A.J. (2014). Atlas of Multiple Sclerosis 2013: A growing global problem with widespread inequity. Neurology.

[3] Dobson R., Giovannoni G. (2018). Multiple sclerosis—A review. Eur. J. Neurol..

[4] Pawlitzki M., Horbrügger M., Loewe K., Kaufmann J., Opfer R., Wagner M., Al-Nosairy K.O., Meuth S.G., Hoffmann M.B., Schippling S. (2020). MS optic neuritis-induced long-term structural changes within the visual pathway. Neurol. Neuroimmunol. Neuroinflamm..

[5] Bennett J.L. (2019). Optic Neuritis. CONTINUUM Lifelong Learn. Neurol..

[6] Redler Y., Levy M. (2020). Rodent Models of Optic Neuritis. Front. Neurol..

[7] Donica V.C., Alexa A.I., Pavel I.A., Danielescu C., Ciapă M.A., Donica A.L., Bogdănici C.M. (2023). The Evolvement of OCT and OCT-A in Identifying Multiple Sclerosis Biomarkers. Biomedicines.

[8] Thompson A.J., Banwell B.L., Barkhof F., Carroll W.M., Coetzee T., Comi G., Correale J., Fazekas F., Filippi M., Freedman M.S. (2018). Diagnosis of multiple sclerosis: 2017 revisions of the McDonald criteria. Lancet Neurol..

[9] Behbehani R., Ali A., Al-Omairah H., Rousseff R.T. (2020). Optimization of spectral domain optical coherence tomography and visual evoked potentials to identify unilateral optic neuritis. Mult. Scler. Relat. Disord..

[10] Halliday A.M., Mcdonald W.I., Mushin J. (1972). DELAYED VISUAL EVOKED RESPONSE IN OPTIC NEURITIS. Lancet.

[11] Berman S., Backner Y., Krupnik R., Paul F., Petrou P., Karussis D., Levin N., Mezer A.A. (2020). Conduction delays in the visual pathways of progressive multiple sclerosis patients covary with brain structure. NeuroImage.

[12] Abel A., McClelland C., Lee M.S. (2019). Critical review: Typical and atypical optic neuritis. Surv. Ophthalmol..

[13] Luo J.J., Bumanlag F., Dun N. (2019). Low-contrast visual evoked potential and early detection of optic demyelination. J. Neurol. Sci..

[14] Yang E.B., Hood D.C., Rodarte C., Zhang X., Odel J.G., Behrens M.M. (2007). Improvement in Conduction Velocity after Optic Neuritis Measured with the Multifocal VEP. Investig. Opthalmol. Vis. Sci..

[15] Oertel F.C., Krämer J., Motamedi S., Keihani A., Zimmermann H.G., Dimitriou N.G., Condor-Montes S., Bereuter C., Cordano C., Abdelhak A. (2023). Visually Evoked Potential as Prognostic Biomarker for Neuroaxonal Damage in Multiple Sclerosis From a Multicenter Longitudinal Cohort. Neurol. Neuroimmunol. Neuroinflamm..

[16] Amezcua L., Robers M.V., Soneji D., Manouvakhova O., Martinez A., Islam T. (2023). Inclusion of optic neuritis in dissemination in space improves the performance of McDonald 2017 criteria in Hispanic people with suspected multiple sclerosis. Mult. Scler. J..

[17] Vidal-Jordana A., Rovira A., Calderon W., Arrambide G., Castilló J., Moncho D., Rahnama K., Collorone S., Toosy A.T., Ciccarelli O. (2024). Adding the Optic Nerve in Multiple Sclerosis Diagnostic Criteria: A Longitudinal, Prospective, Multicenter Study. Neurology.

[18] Roldán M., Caballé N., Sainz C., Pérez-Rico C., Ayuso L., Blanco R. (2024). Assessing the visual afferent pathway with the multifocal visual evoked potentials in the radiologically isolated syndrome. Sci. Rep..

[19] Vecchio D., Barbero P., Galli G., Virgilio E., Naldi P., Comi C., Cantello R. (2023). Prognostic Role of Visual Evoked Potentials in Non-Neuritic Eyes at Multiple Sclerosis Diagnosis. J. Clin. Med..

[20] Nikolic B., Zaletel I., Ivancevic N., Rovcanin B., Pepic A., Samardzic J., Jancic J. (2022). The usefulness of visual evoked potentials in the assessment of the pediatric multiple sclerosis. Eur. J. Paediatr. Neurol..

[21] Filgueiras T.G., Oyamada M.K., Hokazono K., Cunha L.P., Apóstolos-Pereira S.L., Callegaro D., Monteiro M.L.R. (2021). Comparison of Visual Evoked Potentials in Patients Affected by Optic Neuritis From Multiple Sclerosis or Neuromyelitis Optica Spectrum Disorder. J. Neuro-Ophthalmol..

[22] Ekayanti M.S., Mahama C.N., Ngantung D.J. (2021). Normative values of visual evoked potential in adults. Indian J. Ophthalmol..

[23] Donica V.C., Donica A.L., Pavel I.A., Danielescu C., Alexa A.I., Bogdănici C.M. (2024). Variabilities in Retinal Hemodynamics Across the Menstrual Cycle in Healthy Women Identified Using Optical Coherence Tomography Angiography. Life.

[24] Fortepiani L., Foutch B.K., Wilson M.R. (2021). The Effects of Sex, Oral Contraception, and Menstrual Cycle Phase on Intraocular Pressure, Central Corneal Thickness, and Foveal Thickness: A Descriptive Analysis. Vision.

[25] Vander Wall R., Basavarajappa D., Palanivel V., Sharma S., Gupta V., Klistoner A., Graham S., You Y. (2024). VEP Latency Delay Reflects Demyelination Beyond the Optic Nerve in the Cuprizone Model. Investig. Ophthalmol. Vis. Sci..

[26] Papadopoulou A., Pfister A., Tsagkas C., Gaetano L., Sellathurai S., D’Souza M., Cerdá-Fuertes N., Gugleta K., Descoteaux M., Chakravarty M.M. (2024). Visual evoked potentials in multiple sclerosis: P100 latency and visual pathway damage including the lateral geniculate nucleus. Clin. Neurophysiol..

[27] Ciapă M.A., Șalaru D.L., Stătescu C., Sascău R.A., Bogdănici C.M. (2022). Optic Neuritis in Multiple Sclerosis—A Review of Molecular Mechanisms Involved in the Degenerative Process. Curr. Issues Mol. Biol..

[28] Schurz N., Sariaslani L., Altmann P., Leutmezer F., Mitsch C., Pemp B., Rommer P., Zrzavy T., Berger T., Bsteh G. (2021). Evaluation of Retinal Layer Thickness Parameters as Biomarkers in a Real-World Multiple Sclerosis Cohort. Eye Brain.

[29] Tepavčević V., Lubetzki C. (2022). Oligodendrocyte progenitor cell recruitment and remyelination in multiple sclerosis: The more, the merrier?. Brain.

[30] Deschamps R., Shor N., Vignal C., Guillaume J., Boudot de la Motte M., Salviat F., Lecler A., Marignier R., Hage R., Coulette S. (2022). Prospective longitudinal study on prognostic factors of visual recovery and structural change after a first episode of optic neuritis. Eur. J. Neurol..

[31] Carroll W.M., Jennings A.R., Mastaglia F.L. (1990). The origin of remyleinating oligodendrocytes in antiserum-mediated demyelinative optic neuropathy. Brain.

[32] Prineas J.W., Barnard R.O., Kwon E.E., Sharer L.R., Cho E. (1993). Multiple sclerosis: Remyelination of nascent lesions: Remyelination of nascent lesions. Ann. Neurol..

[33] Lucchinetti C.F., Brück W., Rodriguez M., Lassmann H. (1996). Distinct Patterns of Multiple Sclerosis Pathology Indicates Heterogeneity in Pathogenesis. Brain Pathol..

[34] Hanafy K.A., Sloane J.A. (2011). Regulation of remyelination in multiple sclerosis. FEBS Lett..

[35] Wu L., Williams A., Delaney A., Sherman D., Brophy P. (2012). Increasing Internodal Distance in Myelinated Nerves Accelerates Nerve Conduction to a Flat Maximum. Curr. Biol..

[36] Cordano C., Sin J.H., Timmons G., Yiu H.H., Stebbins K., Guglielmetti C., Cruz-Herranz A., Xin W., Lorrain D., Chan J.R. (2022). Validating visual evoked potentials as a preclinical, quantitative biomarker for remyelination efficacy. Brain.

[37] Saridas F., Hojjati F., Alizada S., Lazrak S., Koc E.R., Turan O.F. (2024). Effects of disease-modifying therapies on remyelination in multiple sclerosis; evaluation via visual evoked potential test. Mult. Scler. Relat. Disord..

[38] Hauser S.L., Cree B.A.C. (2020). Treatment of Multiple Sclerosis: A Review. Am. J. Med..

[39] Barton J.L., Garber J.Y., Klistorner A., Barnett M.H. (2019). The electrophysiological assessment of visual function in Multiple Sclerosis. Clin. Neurophysiol. Pract..

[40] Odom J.V., Bach M., Brigell M., Holder G.E., McCulloch D.L., Mizota A., Tormene A.P. (2016). ISCEV standard for clinical visual evoked potentials: (2016 update). Doc. Ophthalmol..

[41] Vidal-Jordana A., Rovira A., Arrambide G., Otero-Romero S., Río J., Comabella M., Nos C., Castilló J., Galan I., Cabello S. (2021). Optic Nerve Topography in Multiple Sclerosis Diagnosis: The Utility of Visual Evoked Potentials. Neurology.

